# Electrospun Polycaprolactone Membranes Loaded with Gentamicin and Nano-Hidroxyapatite for Guided Bone Regeneration

**DOI:** 10.3390/biomedicines13102349

**Published:** 2025-09-25

**Authors:** Ioana-Codruta Mirica, Gabriel Furtos, Véronique Fontaine, Mihaela Vlassa, Petru Pascuta, Ioan Petean, Bogdan Bâldea, Otilia Andercou, Ondine Patricia Lucaciu

**Affiliations:** 1 Department of Oral Health, Iuliu Hatieganu University of Medicine and Pharmacy, 400012 Cluj-Napoca, Romania; mirica_codruta@yahoo.com (I.-C.M.); ondineluc@yahoo.com (O.P.L.); 2 Department of Dental Materials, Raluca Ripan Institute of Research in Chemistry, Babes-Bolyai University, 400294 Cluj-Napoca, Romania; 3 Bioorganic and Macromolecular Chemistry Unit, Faculty of Pharmacy, Microbiology, Université libre de Bruxelles (ULB), 1050 Bruxelles, Belgium; veronique.fontaine@ulb.be; 4 Department of Physics and Chemistry, Technical University of Cluj-Napoca, 400641 Cluj-Napoca, Romania; petru.pascuta@phys.utcluj.ro; 5 Faculty of Chemistry and Chemical Engineering, Babes-Bolyai University, 400028 Cluj-Napoca, Romania; petean.ioan@gmail.com; 6 Department of Prosthodontic Dentistry, Nicolae Testimiteanu State University of Medicine and Pharmacy, 2004 Chisinau, Moldova; bogdanbaldea@gmail.com; 7 Radiobiology and Tumor Biology Department, Oncology Institute Prof. Dr. I. Chiricuţă, 400015 Cluj-Napoca, Romania

**Keywords:** antimicrobial barrier membrane, guided bone regeneration, gentamicin sulfate, nano-hydroxyapatite, electrospinning

## Abstract

**Background/Objectives**: Polymeric barrier membranes (BMs) are usually used in guided bone regeneration to isolate the bone defect from the surrounding tissue, favoring bone apposition. This study proposes a third-generation BM made of polycaprolactone (PCL), loaded with different concentrations of nano-hidroxyapatite (nHAP) and gentamicin (GEN), and fabricated by electrospinning. **Methods**: The mechanical properties of the polymer, together with the fabrication procedure, offer porosity with interconnectivity to permit cell adhesion and proliferation. Bacterial contamination of the BM can induce infection at the bone level, leading to unfavorable clinical outcomes of the regeneration procedure. **Results:** Therefore, BMs have been proposed as carriers for local GEN antibiotic therapy, demonstrating antibacterial properties against *S. aureus*, *S. mutans*, and *P. aeruginosa*, depending on the drug concentration, while being negligibly affected by the nHAP content. X-ray diffraction, FTIR-ATR, and SEM allowed for BM structural characterization, demonstrating the presence of GEN/nHAP and establishing the fiber diameter, which influences the mechanical properties in dry and wet conditions and the drug release behaviorA BM cytotoxicity assessment, performed over 1 and 5 days, revealed that a high nHAP concentration provided protection against cytotoxicity, in contrast to GEN, and that cell proliferation and cell adhesion increased in the presence of nHAP. The BM’s bioactivity was demonstrated by mineralization after 21 days in simulated body fluid in an SEM/EDX analysis. **Conclusions**: The electrospun 15 wt.% nHAP and 2 wt.% GEN-loaded third-generation BM could be a promising alternative for guided bone regeneration.

## 1. Introduction

Dental implants are used worldwide to replace lost teeth [1]. Usually, the limited three-dimensional bone structure leads to the prosthesis being difficult or impossible to implant, increasing the risk of a decrease in quality of life [2]. Guided bone regeneration (GBR) is a surgical procedure often used to overcome bone loss. This procedure requires barrier membranes (BMs) that play an important role by blocking the proliferation of the regenerated connective tissue in the bone defect, ensuring in this way mechanical support for the overlying tissue and providing local drug delivery [3]. Third-generation BMs are the latest ones and act not only as barriers but also as devices releasing therapeutic or bioactive agents in a predefined time to support natural, superior healing of the wound and increase the reconstruction’s chances of success [4]. GBR is performed in combination with autologous bone grafts or allogeneic or xenogenic bone substitutes [2]. The main cause of failure of bone regeneration is represented by infections [5]. Although antibiotics are usually effective, their local concentration can sometimes be too low to fight the infection [6,7]. Furthermore, long-term administration may lead to side effects like drug-induced hepatitis, nephrotoxicity, or myelosuppression [6]. Local administration is generally desired due to the reduced delivery time, high drug concentration at infection sites, and absence of systemic side effects [8]. In addition, overexposure to antibiotics is avoided, reducing the risk of antibacterial resistance [9]. Over the last few years, the electrospinning technique has attracted much interest in the pharmaceutical field for applications such as drug delivery systems. In comparison with other formulations, electrospinning allows for great flexibility in selecting and mixing materials and drugs. Nanoscale formulations, such as nanofibers, have attracted special attention during the last decade [10].

Gentamicin (GEN) sulfate is an aminoglycoside that acts bactericidally against many microorganisms, including *S. aureus* [11], which is known to cause osteomyelitis [12], *S. mutans*, which colonizes the oral cavity, and *P. aeruginosa*, which is often detected in nosocomial infections. It is usually used to prevent or treat bone infections [13]. Polycaprolactone (PCL) is used to develop biomaterials for medical applications because it is easy to process, biocompatible, has good mechanical properties, and yields non-toxic degradation products [14]. Hydroxyapatite (HAP) is widely used for the scaffolds needed in GBR because of its similarity to bone minerals, biocompatibility [15], osteoinductivity [16], and higher mechanical properties than other bioceramics [15]. Despite HAP’s low bioresorbability, this surface can launch nucleating sites for the precipitation of apatite crystals, stimulating cell attachment and growth [15]. Nano-hydroxyapatite (nHAP) is usually used in the development of new bioactive materials because of its large surface area [17]. Different fibrous composite scaffolds have been developed over the last few years for repairing and regenerating bone tissue [18]. New biomaterials loaded with nHAP and different types of antibiotics showed promising *in vitro* results in fighting infections and promoting bone growth [19,20,21,22,23].

The overall aim of this research was to combine two different compounds (nHAP and GEN) in the same fiber of a BM and, by varying their concentration, develop 15 new, third-generation BMs through electrospinning. To the best of our knowledge, the literature does not contain similar materials characterized through the same methods. nHAP was characterized by size, morphology, and purity using different methods. The interaction between nHAP and GEN was evaluated by FTIR. The morphology (determined by SEM, EDX, and AFM), mechanical performance, antibacterial activity (determined by disk diffusion assay, anti-nascent biofilm activity assay, and antibacterial adhesion), biological behavior (cytotoxicity, cell proliferation, and cell adhesion), drug release behavior, and bioactivity of the BMs were studied.

## 2. Materials and Methods

### 2.1. Materials

PCL with a molecular weight (M.W.) of 80,000 g·mol^−1^, poly(vinyl alcohol) (PVA), diammonium hydrogen phosphate ((NH_4_)_2_HPO_4_), calcium nitrate tetrahydrate (Ca(NO_3_)_2_·4H_2_O), ammonium hydroxide solution (NH_4_OH), GEN, methanol, chloroform, Sodium Dodecyl Sulphate (SDS), tryptic soy agar (TSA), a PKH26 Red Fluorescent Cell Linker Kit, 12-well plates, 96-well plates, and Petri dishes were acquired from Sigma-Aldrich, Darmstadt, Germany. Darvan 821A was purchased from R. T. Vanderbilt (Norwalk, CT, USA). Deionized water was used in all experiments. The resistivity of absolute pure water was 18.2 MΩ·cm at 25 °C and the electrical conductivity was 0.055 µS/cm. All commercial materials were used without further purification. The bacterial *S. aureus* ATCC 6538 and *S. mutans* ATCC 25175 strains, the human adherent keratinocyte cell line (HaCaT), and human fibroblasts (BJ CRL-2522™) were obtained from the American Type Culture Collection (ATCC, Manassas, VA, USA). The *P. aeruginosa* LMG 6395 strain was acquired from the BCCM/LMG collection (Ghent, Belgium). The simulated body fluid (SBF) was prepared according to Kokubo’s formulation [24].

### 2.2. Methods

#### 2.2.1. Synthesis of nHAP

Calcium nitrate and ammonium hydrogen phosphate from Ca and P precursors and a wet chemical method were used. In two beaker glasses (4000 mL), two solutions of 2000 mL were prepared by separately mixing and strongly stirring the calcium nitrate and ammonium hydrogen phosphate with double-distilled water at ambient temperature. A mixture solution (0.2 vol.%) of dispersing agent (Darvan 821A/PVA/Sodium Dodecyl Sulphate (SDS) at 1.5:1:1) was added to both solutions. A 25% NH_4_OH solution was used to adjust the pH of both aqueous solutions. Calcium nitrate solution was mixed with ammonium hydrogen phosphate solution in conformity with the standard stoichiometry for pure HAP at a Ca/P ratio of 1.67. Moreover, nHAP was prepared according to the following reaction:

10Ca(NO_3_)_2_·4H_2_O + 6(NH_4_)_2_HPO_4_ + 8NH_4_OH → Ca_10_(PO_4_)_6_(OH)_2_ + 20NH_4_NO_3_ + 20H_2_O (1)

The solution of (NH_4_)_2_HPO_4_ was added dropwise into the Ca(NO_3_)_2_ solution at 75 °C with strong stirring. A pH of 10.5 was maintained using a solution of 25% NH_4_OH. The suspension solution was kept under stirring for 12 h and the obtained precipitate was filtrated and washed three times with double-distilled water and anhydrous ethanol. Finally, the nHAP particles were lyophilized using the Christ alpha 1-4LD Plus model and dried in an oven at 60 °C for 24 h. The powder was crushed and kept at 50 °C in a furnace for 24 h to obtain nHAP dry powder.

#### 2.2.2. Preparation of the Electrospun BMs

**Figure 1:**
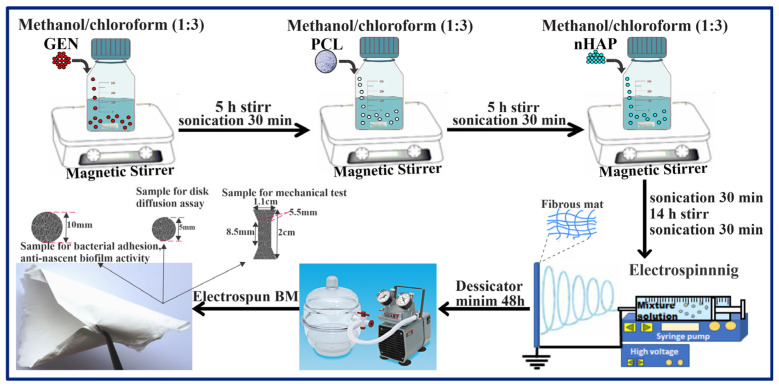
Schematic diagram of general concept of the fabrication of BMs.

A ceramic–drug–polymer solution was prepared via the one-pot procedure using a previously described method [25]. First, GEN was dissolved in a solvent solution of methanol–chloroform (1:3), stirred for 5 h with a magnetic bar, and then sonicated for 30 min. After adding PCL, the mixing process was restarted in the same way. nHAP particles were added to the obtained solution, which was then sonicated for 30 min, stirred for 14 h, and sonicated again for 30 min. For all the obtained BMs, PCL was used in the same quantity (2 g) to yield BMs with a similar thickness. The electrospinning process was performed with an experimental machine from Raluca Ripan, Institute of Research in Chemistry, Cluj-Napoca, Romania. The parameters used were the following: the distance between the tip of the needle and the collector was 31 cm, the flow rate was 2.5 mL/h, the voltage was 15–25 kv, the room temperature was 25 °C, and the humidity was 40% RH. The electrospinning process is sensitive to environmental conditions (room temperature, humidity and pressure) [26]. The obtained BMs (Table 1) were stored in a desiccator for 48 h before testing. A schematic diagram of the general concept for the fabrication of BMs can be seen in Figure 1.

#### 2.2.3. FTIR Spectroscopy, X-Ray Diffraction, TEM, and SEM Analyses

FTIR spectroscopy was carried out in attenuated total reflection mode (ATR). An FTIR spectrophotometer (FTIR-610, Jasco International Co., Ltd., Tokyo, Japan) equipped with an ATR attachment with a horizontal ZnSe crystal was used to collect the spectra. The spectra were scanned in the mid-IR range from 400 to 4000 cm^−1^. The resolution of the spectra was 4 cm, and the scans were repeated 100 times. The spectrum was corrected against the background spectrum. X-ray diffraction was carried out through XRD measurements on a Shimadzu 6000 XRD diffractometer (Kyoto, Japonia), with a monochromator of graphite for the Cu-Kα radiation, at room temperature. The source power was operated at a voltage of 40 kV and a current of 30 mA and the scan speed was 2°/min. The morphology of the BMs was investigated by SEM (SEM Inspect S, FEI, Eindhoven, The Netherlands) using high vacuum. The diameter of nHAP particles from TEM images and the fiber diameter from SEM images were evaluated using Image J software (ImageJ bundled with 64-bit Java 8). After collecting data, the mean diameter and the standard deviation (SD) were calculated and graphs were generated. TEM (H-7650 120 kV automatic microscope, Hitachi, Tokyo, Japan) was used at a voltage of 80 KV to investigate the size and morphology of nHAP particles. The samples were gold-sputtered and analyzed by SEM (SEM Inspect S, FEI, Eindhoven, The Netherlands) using high vacuum.

#### 2.2.4. Mechanical Properties

Samples (n = 5) with a dog-bone shape (gauge width = 5.5 mm, length = 8.5 mm, 0.2 mm thickness), were obtained with an experimental stamp with the shape of a dog bone (Figure 1). The samples were tested in dry and wet conditions, as described in another paper [20], using a mechanical testing machine (LR5K Plus, Lloyd Instruments, Ltd., Bognor Regis, UK) at a loading rate of 1 mm/min.

#### 2.2.5. Microbiological Evaluation of Antibacterial Activities of the BMs

The GEN-loaded BMs were tested for an antibacterial response by the disk diffusion assay on different bacterial strains (*S. aureus* ATCC 6538, *S. mutans* ATCC 25175, *P. aeruginosa* LMG 6395) as described in another paper [20]. An anti-nascent biofilm activity assay (10 mm diameter, 0.2 mm thickness) containing different concentrations of GEN and nHAP, a positive control (a 5-cent coin), and a negative control (glass) was run using *S. aureus* ATCC 6538 with persistent microbial inoculum contact [27]. The samples were incubated for 24 h at 37 °C in the bacterial inoculum suspension with a density of 1 McFarland. After incubation, 7 serial dilutions (1/10) of the bacterial suspension were performed, starting with a first step of inactivation using a neutralization solution (2.57% of Letheen medium, 3.5% polysorbate 80, 0.33% lecithin, 0.1% L-histidine, and 0.4% sodium lauryl sulfate) for the dilution. The next dilutions were performed in buffered peptone solution at a pH of 7. A quantity of 800 µL of the last 4 dilutions was plated in depth in liquefied TSA and, after solidification, incubated for 24 h at 37 °C. The colony forming units (CFU) were quantified on the plates, showing 30 to 300 CFU, and the obtained numbers were converted to CFU/mL depending on the dilution factor used to obtain the observed CFU plates [27]. All the tests were run in triplicate.

The antibacterial adhesion assay used BM disks (10 mm diameter, 0.2 mm thickness) with different concentrations of GEN and the same content of nHAP (15%), which were incubated for 24 h in 1 mL of the bacterial suspension with a density of 1 McFarland at 37 °C for biofilm formation. After this period, samples were rinsed with 1 mL of phosphate-buffered saline (PBS) before bacterial detachment by mechanical force performed in an inactivating solution (the same used above to neutralize any released antimicrobial compounds). The surviving bacteria (microorganisms previously adhering to the surfaces) were enumerated by CFU plate counting of serial sample dilutions [27]. All the tests were run in triplicate.

#### 2.2.6. Cytotoxicity, Cell Proliferation, and Cell Adhesion Assay

The samples were sterilized by β irradiation at 25 Gy for 15 min before being incubated (for 1 day and 5 days at 37 °C) in cell culture medium (DMEM supplemented with 10% fetal bovine serum, penicillin (100 U/mL), and streptomycin (100 µg/mL)) at a concentration of 0.1 g/mL according to ISO 10993 [28]. The collected media were directly used for the tests. The cytotoxic effect of the developed BMs was tested on the human HaCaT keratinocyte cell line in an MTT assay [25]. The cell viability was assessed by measuring the absorbance at 570 nm and 630 nm in each well using a microplate reader (Bio-Rad 680, Bio-Rad, Hercules, CA, USA) and calculated according to Equation (2).

(2) $$Cell\;viability\;\left(\%\right)=\frac{Absorbance\;of\;the\;scaffold-Absorbance\;of\;the\;medium\;control}{Absorbance\;of\;the\;control-Absorbance\;of\;the\;medium\;control}\times 100$$

The cell proliferation and cell adhesion assays were run at 24 h, 72 h, and 7 days on human fibroblasts, which were stained with a membrane linker (PKH26 Sigma Aldrich, Burlington, MA, USA) that is also a viability indicator and is fluorescent. The cells were visualized with a Zeiss microscope (Zeiss GmbH, Jena, Germany) in inverted phase at a wavelength of 546 nm. The method used has been described in detail in another paper [29]. For the cell adhesion assay, the samples were fixed overnight with 4% paraformaldehyde and an SEM analysis was performed. All the tests were run in triplicate.

#### 2.2.7. Drug Release

Samples (n = 3) of BMs loaded with GEN (Table 1) with a weight of 0.025 g were used for the drug release test, which was run at 1, 3, 6, 10, and 18 h using a method published in another work [30].

#### 2.2.8. *In Vitro* Bioactivity

The apatite-forming property (mineralization) of nHAP-loaded BMs was evaluated on samples (width = 4 mm, length = 4 mm, 0.2 mm thickness) incubated at 37 °C for 21 days using a previously described method [20]. Finally, apatite formation in samples was studied after gold sputtering using SEM-EDX at 15 kV and AFM. The AFM investigation was realized with a JSPM 4210 JEOL Scanning Probe Microscope (Jeol, Tokyo, Japan) using tapping mode (intermittent contact) and 15 NSC cantilevers produced by MikroMasch Co. (Sofia, Bulgaria) with a resonant frequency of 325 kHz and a force constant of 40 N/m. The fine microstructure of the mineralized BMs was investigated in a scanned area of 20 μm × 20 μm and the nanostructure was investigated in a scanned area of 2 μm × 2 μm. At least 3 different macroscopic zones on the surface of the samples were scanned. The images were analyzed with Win SPM 2.0 soft powered by JEOL, Tokyo, Japan, providing the fiber diameter and surface roughness parameters (Ra and Rq). At least five determinations were effectuated for each parameter for a proper statistical representation.

#### 2.2.9. Statistical Analysis

Statistical analysis was performed using the SPSS Statistics package (Version 11.5, SPSS Inc., Chicago, IL, USA). The fiber diameter and mechanical properties were determined by one-way analysis of variance (ANOVA) and by Tukey’s test with the level of significance set at 0.05 to determine the significant differences between the mean values of the tested BMs. The cytotoxicity values and the antimicrobial activity were statistically analyzed with the *t*-test. The results obtained were processed to calculate the average value and the standard deviation and to generate graphs. All tests were run three times in triplicate.

## 3. Results and Discussion

**Figure 2:**
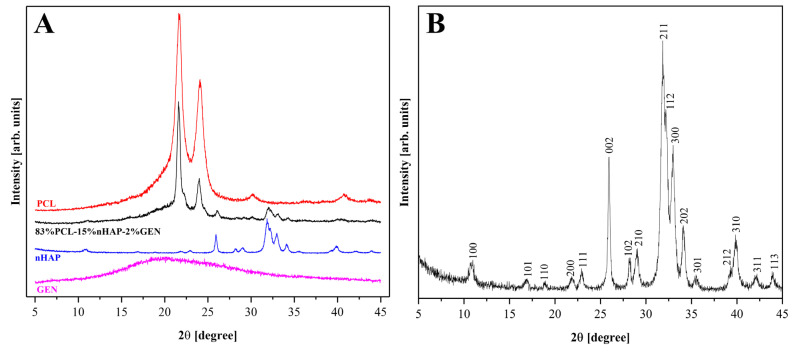
(**A**) XRD analysis of PCL-15%nHAP-2%GEN and (**B**) XRD pattern of nHAP.

**Figure 3:**
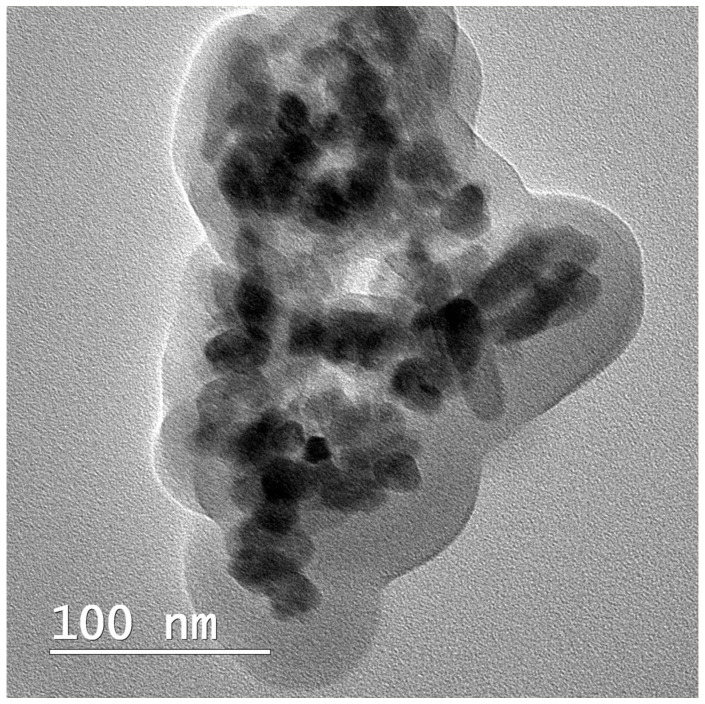
TEM image of the synthesized nHAP powder.

The average crystallite of the nHAP from the XRD measurement (Figure 2A) showed a size of 35.3 nm. The characteristic peaks of nHAP were identified and assigned based on a standard JCPDS PDF card (no. 09-0432). The main diffraction peaks observed at approximately 2θ = 25.9°, 31.8°, 32.9°, and 34.1° are associated with the (002), (211), (112), and (300) planes, confirming the presence of HAP and the absence of additional crystalline phases. The XRD patterns for the BM with the composition 83%PCL-15%nHAP-2%GEN and for all precursors used in the synthesis of the BMs (PCL, nHAP, and GEN) are shown in Figure 2A. The diffractogram (Figure 2A) of the studied BMs demonstrates XRD crystalline features like those of the precursors and the position of the diffraction peaks is approximately the same as that of the precursors. This result indicates no formation of a new crystal phase for the BMs. The TEM investigation (Figure 3) of crystals of nHAP showed a mixture of rod and pseudo-spherical particles with a medium size of 22.22 ± 5.71 nm.

**Figure 4:**
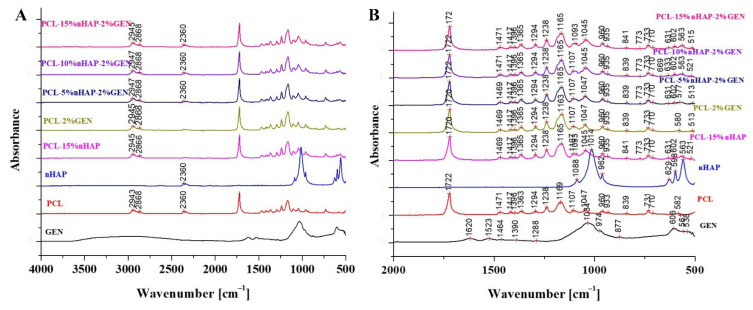
(**A**,**B**) FTIR spectra of GEN, PCL, nHAP, PCL-15%nHAP, PCL-2%GEN, PCL-5%nHAP-2%GEN, PCL-10%nHAP-2%GEN, and PCL-15%nHAP-2% GEN.

The FTIR spectra of the different precursors (PCL, nHAP, and GEN) and polymer matrices (PCL-15%nHAP, PCL-2%GEN, PCL-5%nHAP-2%GEN, PCL-10%nHAP-2%GEN, and PCL-15% nHAP-2%GEN) are shown in Figure 4A,B. The FTIR spectra of GEN sulfate (Figure 4A,B) show the typical absorption bands at 1620, 1523, and 1288 cm^−1^, assigned to the amide I, amide II, and amide III bond of GEN, respectively [31]. The peak observed at 1034 cm^−1^ was due to the HSO_4_^−^ group, and the one at 606 cm^−1^ was due to the SO_2_ band [31]. GEN contains four major components. The major and minor components (Figure 4A,B) are often positional isomers, have the same backbone, and differ slightly in terms of chemical structure and spectroscopic properties [28]. The PCL spectrum allowed for the identification of the absorption bands at 2943 cm^−1^, attributed to asymmetric CH_2_ stretching, and at 2866 cm^−1^, attributed to symmetric CH_2_ stretching [32]. At 1722 cm^−1^ a strong band, corresponding to the carbonyl (C=O) stretching mode, was identified. The band at 1240 cm^−1^ was identified as asymmetric C=O=C stretching and that at 1169 cm^−1^ as symmetric C=O=C stretching [32]. The FTIR spectrum of nHAP shows the presence of a high peak found at 1014 cm^−1^, attributed to the non-degenerate symmetric stretching of the phosphate groups. Also, characteristic bands for PO_4_^3−^ appear at 1088, 962, 598, and 563 cm^−1^. Thus, the band at 962 cm^−1^ was attributed to the asymmetric P-O stretching vibration of the PO_4_^3−^ and the bands at 629, 598, and 563 were assigned to the triply degenerate bending vibration of PO_4_^3−^ in HAP [33]. Some characteristic peaks for GEN were found to overlap peaks obtained with the PCL and nHAP in the drug–polymer mixture. No chemical bond was formed between GEN, nHAP, and the PCL matrix. The spectra of polymers and the specific bands of GEN are highlighted in Figure 4B. Values of the absorption bands of GEN, such as amide III at 1288 cm^−1^ were overlapped by the 1294 cm^−1^ band of PCL, and the 1034 cm^−1^ band (non-degenerate symmetric stretching of the phosphate groups) and did not show any displacement after being introduced into the polymeric BM.

**Figure 5:**
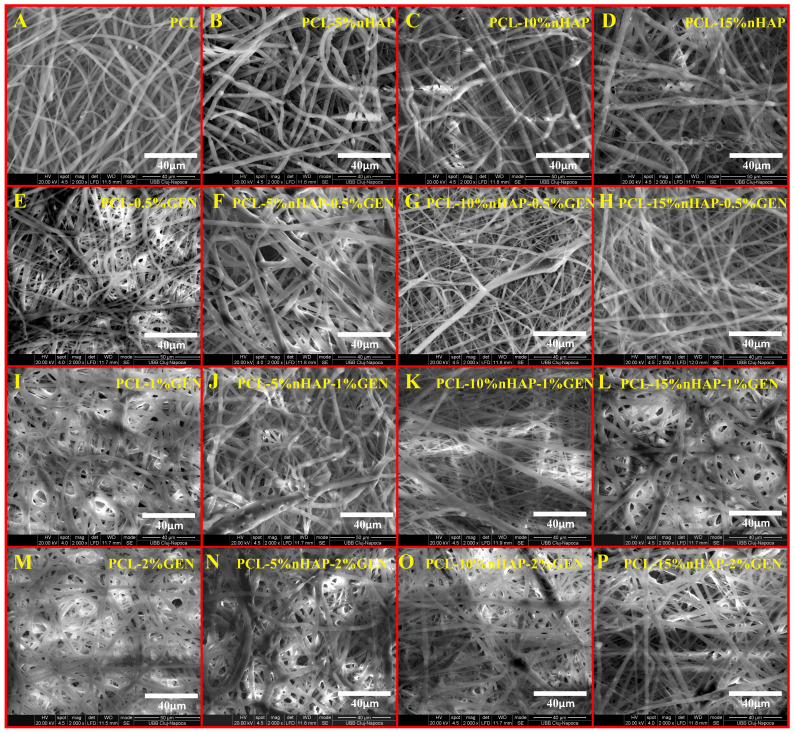
SEM micrographs of electrospun membranes. (**A**) PCL; (**B**) PCL-5%nHAP; (**C**) PCL-10%nHAP; (**D**) PCL-15%nHAP; (**E**) PCL-0.5%GEN; (**F**) PCL-5%nHAP-0.5%GEN; (**G**) PCL-10%nHAP-0.5%GEN; (**H**) PCL-15%nHAP-0.5%GEN; (**I**) PCL-1%GEN; (**J**) PCL-5%nHAP-1%GEN; (**K**) PCL-10%nHAP-1%GEN; (**L**) PCL-15%nHAP-1%GEN; (**M**) PCL-2%GEN; (**N**) PCL-5%nHAP-2%GEN; (**O**) PCL-10%nHAP-2%GEN; (**P**) PCL-15%nHAP-2%GEN.

**Figure 6:**
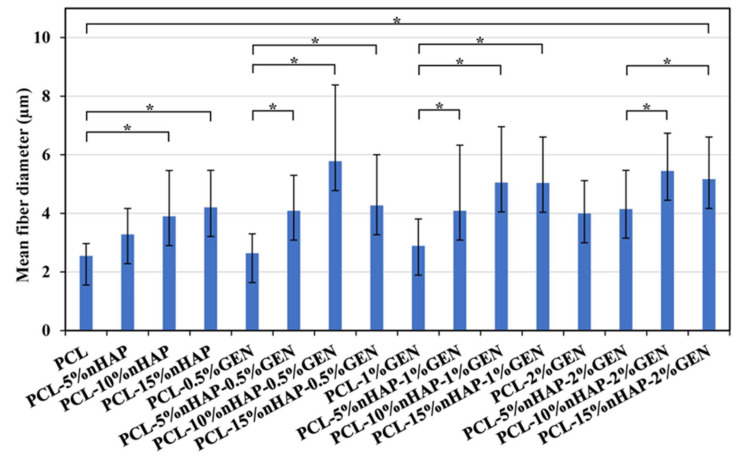
Fiber diameter from SEM images of BMs, using Tukey’s test (* *p* < 0.05) (only the most representative BMs are shown in the figure; detailed statistics are given in Table S1).

The SEM analysis of the BMs revealed randomly aligned bead-free fibers layer by layer, with a typical electrospun structure, highly anisotropic, which formed interconnected pores with different sizes (Figure 5), like other studies [20,32]. The size of the fibers increased as the nHAP content increased, being not influenced by the GEN content (Figure 5), like in another study [34]. The diameter of the fibers ranged from 2.55 µm for the unloaded BM (PCL) to 4.21 µm for the PCL-15%nHAP BM. The PCL-15%nHAP-2%GEN BM showed fibers with a mean diameter of 5.17 µm (Figure 6).

**Figure 7:**
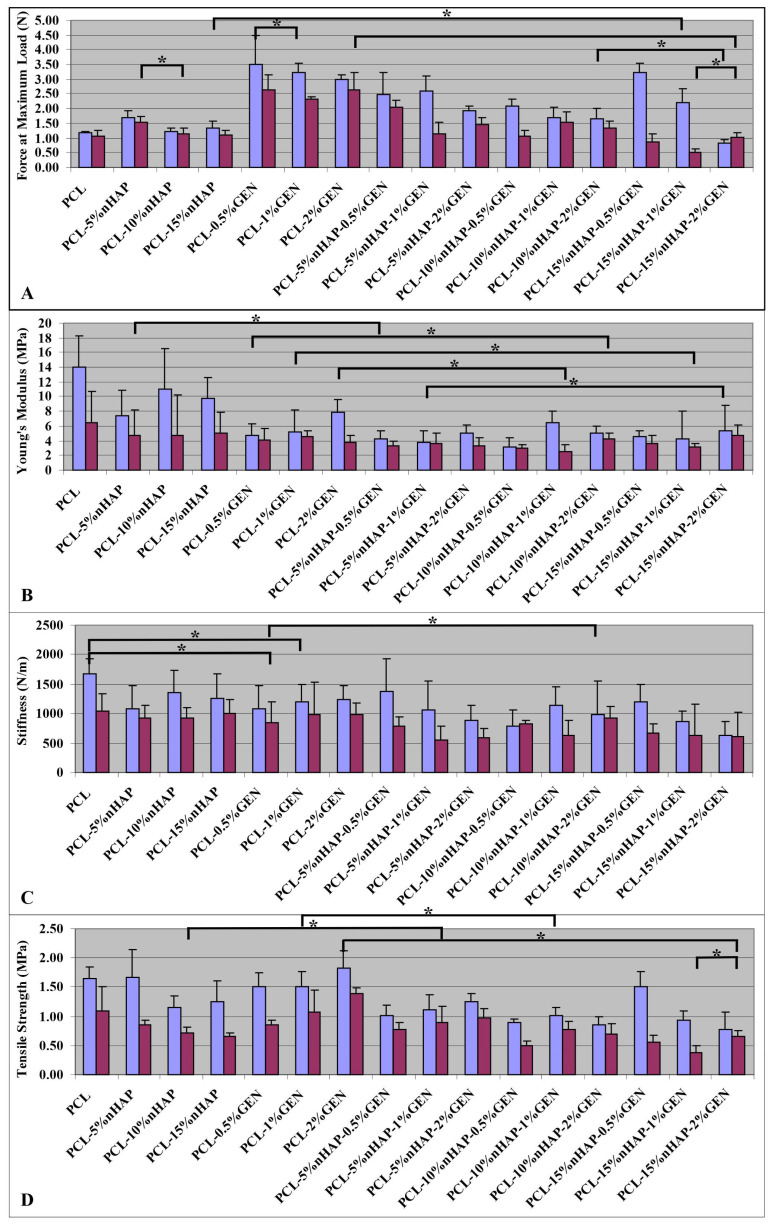
Mechanical properties of BMs before (light blue column) and after 12 h of immersion in SBF (violet column). (**A**) Force at maximum load (N); (**B**) Young’s modulus (MPa); (**C**) stiffness (N/m); and (**D**) tensile strength (MPa), with statistically significant differences using Tukey’s test (* *p* < 0.05) (only the most representative BMs are shown in the figure; detailed statistics are given in Tables S2–S5).

The results on the mechanical properties of the evaluated BMs (Figure 7) show lower mechanical properties in wet conditions than in dry conditions (Figure 7). This behavior could be explained by the plasticization effect of the water and agrees with another study [20]. The force at maximum load (Figure 7A) showed a small increase when nHAP was added from 5% to 15 wt.% to the PCL and a decrease by the inclusion of only GEN from 0.5% to 2 wt.%. All BMs showed a decrease by the addition of both nHAP and GEN. Young’s modulus showed the highest value for PCL compared with other BMs with nHAP or GEN or both because they are mechanically embedded in the PCL fiber. The addition of nHAP from 5% wt. to 10% wt. increased Young’s modulus and a smaller decrease was observed at 15% wt. This means that 10% wt. could be the limit for Young’s modulus for BMs with nHAP. This parameter decreased when GEN was added (Figure 7B), a fact that could be explained by the higher mechanical properties of nHAP compared with GEN. This result supports the hypothesis that the presence of GEN leads to an increase in the mechanical properties.

BM stiffness is an important property because BMs must withstand the weight of the overlying tissue during the healing period [35]. In our study, the highest stiffness was obtained for PCL and there were no statistical differences when nHAP or GEN was added to the PCL fibers (Figure 7C). An explanation could be the missing chemical bond between PCL, nHAP, and GEN because the precursors GEN and nHAP are embedded mechanically in the PCL fibers.

The results for the TS test show values between 0.77 and 1.82 MPa in dry conditions and 0.37 and 1.38 MPa in wet conditions. The TS value increased when 5 wt.% nHAP was added and decreased slowly at higher nHAP contents. This could be considered the limitation of the material. The addition of nHAP in 5%, 10%, and 15% wt. decreased the TS (Figure 7D). PCL-5%nHAP had a higher value than PCL. In contrast to GEN 0.5%, 1% to 2% increased the TS, and PCL-0.5% GEN showed the highest value among all obtained BMs. This can be attributed to the fact that nHAP exhibits increased stiffness compared with GEN, reducing the possibility of fiber stretching. The fibers containing nHAP were even thicker than those with GEN, a fact confirmed by SEM analysis (Figure 5 and Figure 6). When the percentage of nHAP was kept constant at 5% wt. nHAP and the GEN amount varied between 0.5%, 1%, and 2% wt., the TS increased. At the addition of 10% wt. nHAP and fluctuating the GEN amount between 0.5% and 1% wt., the TS increased, and at 1% wt. showed a slow decrease. For the addition of 15% wt. nHAP and modifying the GEN amount between 0.5%, 1%, and 2% wt., we observed a slow decrease in the TS. PCL-15%nHAP-0.5% GEN presented the highest TS values of all BMs with nHAP and GEN but had lower TS values than PCL-2% GEN, PCL-5%nHAP, and PCL (Figure 7D). All BMs tested in dry conditions proved to have higher TS values than in wet conditions. Overall, the mechanical properties of our obtained BMs are similar to the commercial ones that we use in clinical practice [36].

The antibacterial activity of the developed BMs was tested on three different bacterial strains. *S. aureus* was among our choices because it is the most common bacterial strain in implant-related infections and adult osteomyelitis, but also because of its capacity to infect the bone matrix [37,38] and deform and penetrate (sub-)micron structures *in vitro* [39]. This could explain the high recurrence rate of osteomyelitis as bacteria residing in bone canaliculi can escape local and systemic antibiotic therapies without causing tissue necrosis and be easily missed during surgical debridement procedures [40]. We used *S. mutans* in our tests because it colonizes the oral cavity and can easily contaminate the BM during the surgical procedure [41]. We used *P. aeruginosa* as it is one of the most common Gram-negative bacteria in nosocomial infections [42].

**Figure 8:**
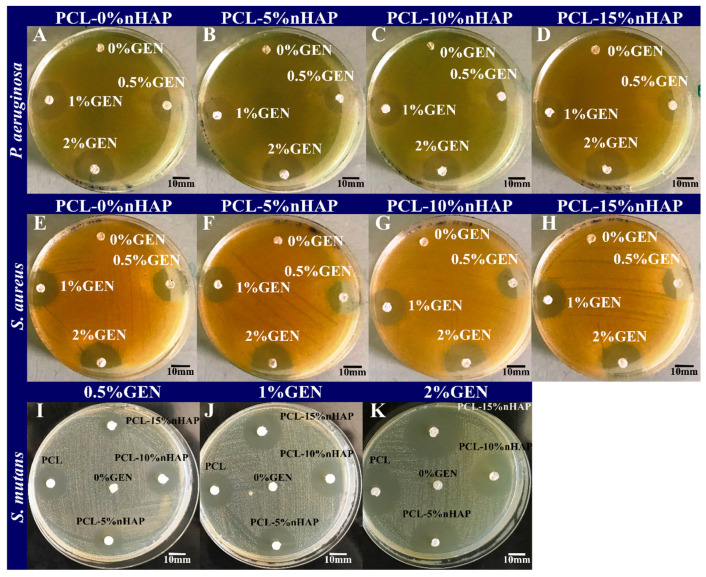
Disk diffusion assay on *P. aeruginosa* (**A**–**D**), *S. aureus* (**E**–**H**), and *S. mutans* (**I**–**K**).

**Figure 9:**
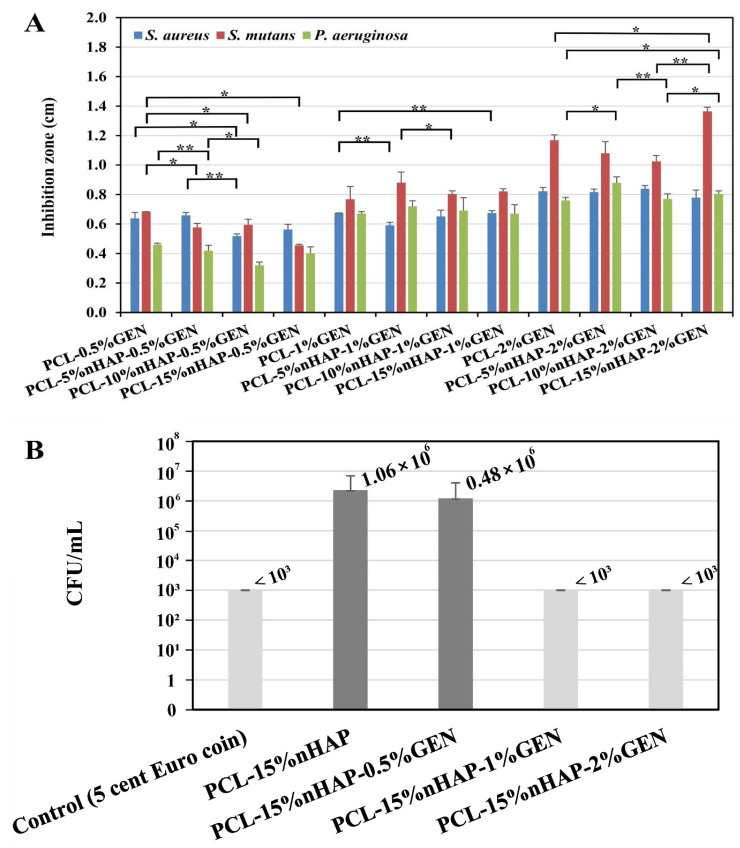
Antibacterial behavior. (**A**) Inhibition zones of the GEN-loaded BM (* *p*-value *<* 0.05; ** *p*-value < 0.01); (**B**) antibacterial activity of the BM on the *S. aureus* anti-nascent biofilm.

The antibacterial activities of the BMs were first assessed by the disk diffusion assay (Figure 8). The growth inhibition zones were 0.64–0.84 cm in diameter for *S. aureus*, between 0.68 and 1.36 cm for *S. mutans,* and between 0.46 and 0.88 cm for *P. aeruginosa* (Figure 9A). As expected, the size of the inhibition zone was correlated with the amount of GEN and was negligibly influenced by the nHAP load (Figure 9A). The most susceptible bacterial strain to GEN was *S. mutans*, and the least susceptible strain was *P. aeruginosa*, as previously reported [43,44]. As expected, the presence of nHAP did not influence the antibacterial activity of the BMs.

*S. aureus* is partly dangerous because of its capacity to form biofilms on different implants and medical devices [45]. Figure 9B shows that PCL with concentrations of 1% and 2 wt.% GEN (even 0.5 wt.%) can inhibit the growth of *S. aureus*, as previously observed [46]. This growth inhibition was also observed in an anti-nascent biofilm activity assay (Figure 9B). Similarly to the assay performed by agar diffusion, PCL with 0.5% GEN was less antibacterial, with less than a 1-log reduction, than PCL with 1% or 2 wt.% GEN, which showed at least a 3-log reduction (bacterial survival was below the detection limit of the test).

**Figure 10:**
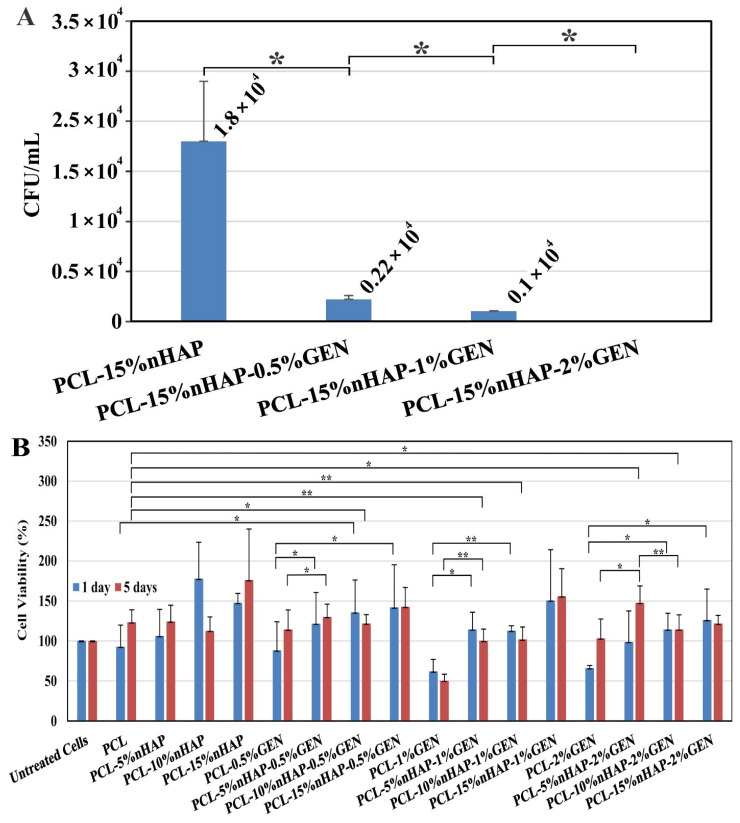
Antibacterial and cytotoxic behavior: (**A**) bacterial adhesion of *S. aureus* on different GEN-loaded BMs (* *p*-value < 0.05); (**B**) cell viability after 1 and 5 days of exposure to the BM, investigated on HaCaT cells in an MTT assay (* *p*-value < 0.05; ** *p*-value < 0.01).

Bacterial adhesion is known to be one of the reasons for the failure of artificial implants in the human body [47]. To verify that the antibacterial activity of the PCL-GEN could also reduce the binding of bacteria to a support, we investigated the impact of the PCL-15%nHAP BM with different concentrations of GEN on bacterial adhesion. As shown in Figure 10A, PCL containing GEN could reduce *S. aureus* adhesion, depending on the GEN concentration. In the presence of 2 wt.%. GEN, we could not even detect adherent *S. aureus* survival. These results agree with the anti-nascent biofilm activities of the BM (Figure 9B).

In addition to good mechanical properties and antibacterial properties, the developed BM must be biocompatible and have no cytotoxicity. The BM must favor the growing and multiplication of cells while maintaining structural integrity [48]. Our results concerning the cytotoxicity of the developed BMs were obtained by running the MTT assay on the HaCaT cell line, a non-tumorigenic immortalized human keratinocyte cell line, for 1 and 5 days (Figure 10B). After 1 day, the addition of nHAP increased the cell viability while the addition of GEN reduced the viability of the cells (Figure 10B). On the other hand, the addition of 10 wt.% nHAP increased the cell viability to 177.93% by enriching functional proteins, stimulating cell proliferation [49], and counteracting GEN cytotoxicity, which acts on the mitochondria to induce apoptosis [50], allowing the obtained BM to release GEN without cytotoxicity.

**Figure 11:**
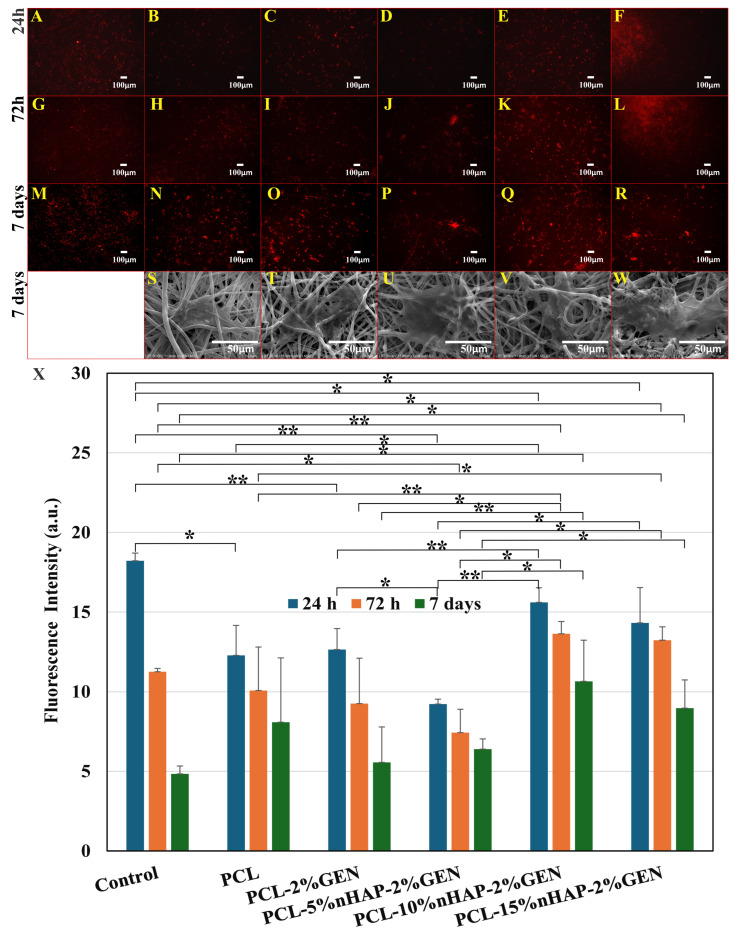
Cell proliferation at 24 h: (**A**) Control; (**B**) PCL; (**C**) PCL-2%GEN; (**D**) PCL-5%nHAP-2%GEN; (**E**) PCL-10%nHAP-2%GEN; (**F**) PCL-15%nHAP-2%GEN. At 72 h: (**G**) Control; (**H**) PCL; (**I**) PCL-2%GEN; (**J**) PCL-5%nHAP-2%GEN; (**K**) PCL-10%nHAP-2%GEN; (**L**) PCL-15%nHAP-2%GEN. At 7 days: (**M**) Control; (**N**) PCL; (**O**) PCL-2%GEN; (**P**) PCL-5%nHAP-2%GEN; (**Q**) PCL-10%nHAP-2%GEN; (**R**) PCL-15%nHAP-2%GEN. SEM images of cell attachment: (**S**) PCL; (**T**) PCL-2%GEN; (**U**) PCL-5%nHAP-2%GEN; (**V**) PCL-10%nHAP-2%GEN; (**W**) PCL-15%nHAP-2%GEN. (**X**) Fluorescence map (* *p*-value < 0.05; ** *p*-value < 0.01).

This could be observed not only after 1 day of exposure but also after 5 days of exposure (Figure 10B). The effect of nHAP on cell growth stimulation has previously been reported [51] and was further investigated by seeding human fibroblasts on the BMs for 24 h, 72 h, and 7 days (Figure 11A–R). The obtained results show that the presence of nHAP influences the proliferation of the cells (Figure 11F,L) (a fact also proved by the MTT assay), except for PCL-2% GEN-5%nHAP, where a drop in the cell proliferation at 24 h can be seen. At 7 days, the proliferation rate for the BM with 15%nHAP was lower than the one for the BM loaded with 10%nHAP (Figure 11Q,R). All these findings are sustained by the fluorescence map (Figure 11X) and show that the addition of nHAP to PCL improves the hydrophobicity of the polymer, allowing the cells to attach and proliferate, like in another study [52]. The SEM images showed that cell attachment occurred along the electrospun fiber (Figure 11S–W). This finding is like that of another study [53]. The cell behavior on scaffolds is shown in Figure 11, and, as can be seen, a cell layer almost formed on PCL-2%GEN-15%nHAP, in contrast to the unloaded PCL BM. Based on these preliminary cell experiments, it can be concluded that the PCL-nHAP BM provides more cell binding sites than the PCL-GEN BM, like in another study [54], with the exception of PCL-2%GEN-5%nHAP and PCL-2%GEN-15%nHAP.

**Figure 12:**
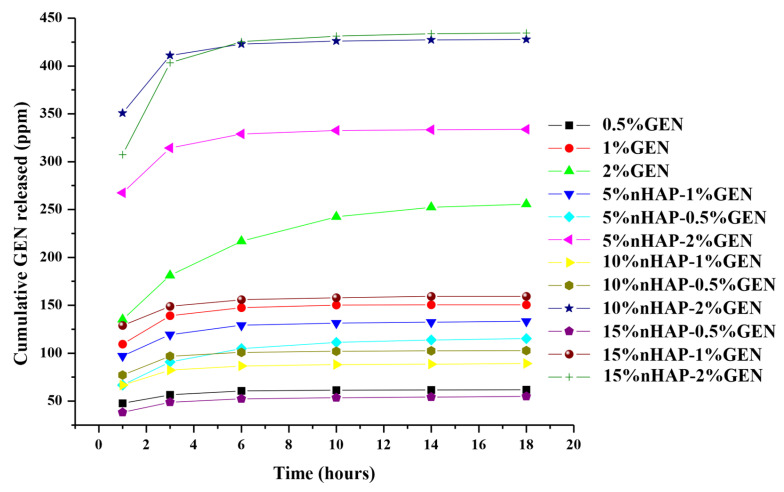
*In vitro* GEN release profiles from BMs.

An alternative way to enhance local antibiotic delivery is to load antibiotics into carrier constructs that can release drugs over a suitable period, in an optimal concentration, and in the proximity of the infection site [55]. A previous study reported that around 80 wt.% of the drug quantity should be released in 7 days [40]. The cumulative release profiles of GEN from the prepared BM, shown in Figure 12, present two phases (an initial rapid release phase, usually considered to be due to a “burst” effect, followed by a slow and prolonged second phase), also observed in another study [20]. The most important is the quantity released in the first phase because the goal is to inhibit rapid bacterial growth or survival before biofilm development. The low quantities from the second phase do not have such an important effect but will contribute to maintaining the reduction in microorganism survival. In our study, we observed an increase in the quantity of GEN released in correlation with the amount of GEN in the BM. The BM with 2 wt.% showed the highest GEN release, as expected. Another observation was the fact that an increase in the addition of nHAP increased the GEN release for the BM with 2 wt.%. GEN. This can be explained by the missing chemical bond between PCL, nHAP, and GEN and the possibility of water penetrating the bulk material more easily. The water solubility of GEN and the water absorption of the PCL matrix allowed for rapid dissolution and increased the GEN release rate. The highest GEN release rate from our BMs was observed for PCL-10%nHAP-2%GEN, which released 434.51 ppm within the first 6 h. Therefore, within the first 6 h, 54.86 wt.% of the total GEN quantity can be released in the proximity of the BM. Consequently, of all the GEN-loaded BMs, we consider this BM to be the most promising for further studies, like *in vivo* studies.

**Figure 13:**
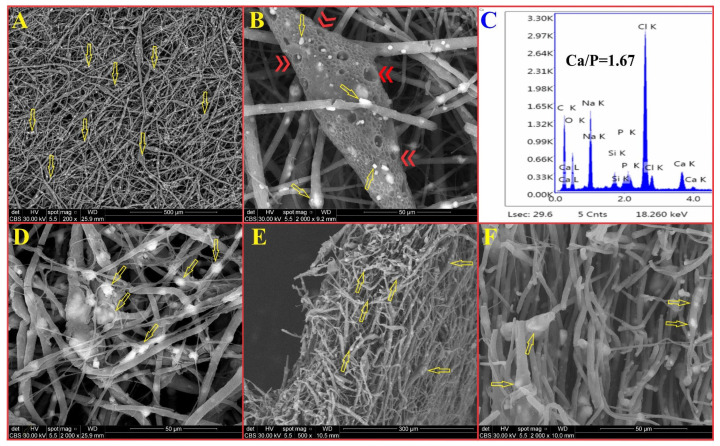
Biomineralization of the BM PCL-15%nHAP-2%GEN. (**A**,**B**,**D**) Biomineralization at the surface (yellow arrows indicate new apatite crystals that formed at the surface of nanofibers from membranes, red double arrows indicate pores inside the fibers); (**C**) EDX analysis; (**E**,**F**) fractured biomineralized membranes.

The addition of nHAP to a BM is expected to raise the BM’s bioactivity, a property required to form a bone-bonding interface between the bone tissue and the BM [55]. SEM micrographs of the surface of the PCL-15%nHAP-2%GEN BM after 21 days of storage in SBF are presented in Figure 13A–F. Many irregular white particles that formed at the surface of nanofibers (yellow arrows) were noticed (Figure 13A–D). Following the EDX analysis, we associated these white particles with the formation of new apatite crystals (Figure 13C), in agreement with a previous study [20]. During the 21 days in SBF, the solution penetrated the PCL fiber loaded with nHAP and induced apatite nucleation and crystallization. The solubility of nHAP particles from the surface of the PCL fibers and the release of Ca^2+^ and HPO_4_^2−^ ions in the SBF induced an increase in ion saturation in the solution. Progressively, a new combination of ions occurred, and the formation of apatite crystals was observed at a Ca/P ratio of 1.67 (Figure 13C). Red double arrows (Figure 13B) indicate microporosities, which may be associated with PCL degradation around the nHAP/GEN particles and/or with the solubility/leaching of nHAP/GEN particles during the storage period. These changes have previously been reported [20]. In Figure 13E,F, the SEM image of a fractured biomineralized BM shows the porosity and higher degree of biomineralization inside of the BM (yellow arrows). These results confirm the bioactivity of our developed BM [56].

**Figure 14:**
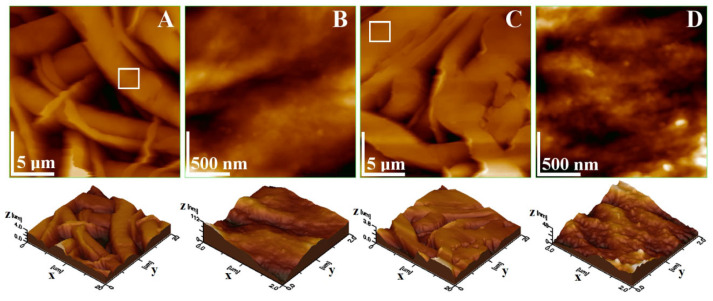
AFM topographic images of the mineralized PCL-15%nHAP-2%GEN BM. (**A**) F1 fine microstructure; (**B**) F1 nanostructure; (**C**) F2 fine microstructure; and (**D**) F2 nanostructure. Three-dimensional profiles are displayed under each topographic image. (**B**,**D**) area the magnified version of the white square areas in (**A**,**C**).

Mineralized PCL-15%nHAP-2%GEN samples obtained after 21 days of storage in SBF were topographically studied through AFM investigation. The fine microstructure of F1 NM presented an interlaced texture of larger fibers, having a diameter of about 4.82 ± 0.22 μm, and smaller fibers with a diameter of about 1.54 ± 0.08 μm (Figure 14A). Their texture was compact, ensuring good material cohesion and presenting roughness parameters of Ra = 874.6 ± 32.57 nm and Rq = 1048.4 ± 122.13 nm. The nanostructural detail presented in Figure 14B shows nHAP particles embedded in the PCL matrix, having an average diameter of 83.5 ± 8.4 nm. The smoothness of the mineralization process over the fiber’s surface was sustained by the nano roughness values of Ra = 28.96 ± 4.35 nm and Rq = 38.76 ± 6.12 nm. The fine microstructure topography of F2 shown in Figure 14C evidences well-interlaced fiber bundles, with larger fibers having a diameter of about 7.0 ± 0.47 μm and several narrow fibers with a diameter of around 1.93 ± 0.08 μm. This observation demonstrates the better compactness of the fine microstructure, which leads to slightly lower roughness values (Ra = 320 ± 75 nm and Rq = 440.33 ± 82 nm). The HAP nanoparticles are more visible in the F2 nanostructure shown in Figure 14D. They are well embedded into the PCL matrix and present an average diameter of 76.66 ± 6.75 nm. Thus, the nano roughness values have an Ra = 13.06 ± 3.15 nm and an Rq = 17.20 ± 4.08 nm.

Analyzing all obtained results for all 15 BMs, the composition that could achieve our goal in clinical trials is PCL-15%nHAP-2%GEN, which also showed great potential *in vivo* [57].

As a limitation to our study, we acknowledge the fact that bone-forming cells, like osteoblasts, were not used in the biological test and some characteristics of our BMs, like the density, were not studied. We acknowledge these are important tests for BMs and will run them in the future.

## 4. Conclusions

XRD and FTIR-ATR analyses showed the presence of PCL, nHAP, and GEN in the BMs without chemical bonds between them. Furthermore, SEM images confirmed the fibrous and porous structure of all the BMs. The investigated mechanical properties decreased with the addition of nHAP and GEN when tested in dry and wet conditions. All BMs with >0.5 wt.% GEN showed optimal antibacterial behavior. Regarding cytotoxicity, the MTT assays showed that GEN toxicity could be countered by adding nHAP. Cell proliferation and adhesion increased in the presence of nHAP, except for in the PCL-2%GEN-5%nHAP and PCL-2%GEN-15%nHAP BMs. The drug release assay showed that GEN was released in two phases, the first phase representing the burst release phase. The biomineralization potential of the BMs was demonstrated inside and on the surface of samples. The obtained results on the BMs classify them as third-generation BMs. Even PCL-15%nHAP-2%GEN showed lower mechanical properties than the other BMs tested. Taken together, our results suggest that this BM exhibits promising potential but requires further *in vitro* and *in vivo* studies before clinical application.

## Figures and Tables

**Table 1:** The obtained BMs.

No.	Code	Composition of the BM		
		PCL (wt.%)	nHAP (wt.%)	GEN (wt.%)
1	PCL	100	0	0
2	PCL-5%nHAP	95	5	0
3	PCL-10%nHAP	90	10	0
4	PCL-15%nHAP	85	15	0
5	PCL-0.5%GEN	99.5	0	0.5
6	PCL-1%GEN	99	0	1
7	PCL-2%GEN	98	0	2
8	PCL-5%nHAP-0.5%GEN	94.5	5	0.5
9	PCL-10%nHAP-0.5%GEN	89.5	10	0.5
10	PCL-15%nHAP-0.5%GEN	84.5	15	0.5
11	PCL-5%nHAP-1%GEN	94	5	1
12	PCL-10%nHAP-1%GEN	89	10	1
13	PCL-15%nHAP-1%GEN	84	15	1
14	PCL-5%nHAP-2%GEN	93	5	2
15	PCL-10%nHAP-2%GEN	88	10	2
16	PCL-15%nHAP-2%GEN	83	15	2

## Data Availability

Data presented in this study is contained within the article and Supplementary Material.

## Supplementary Materials

The following supporting information can be downloaded at: https://www.mdpi.com/article/10.3390/biomedicines13102349/s1, Table S1: Fiber diameter from SEM images of BM, with statistically significant difference from each other, using Tukey’s test (*p* < 0.05); Table S2: Mechanical properties of BM before and after 12 h immersion in SBF: Force at maximum load (N) with statistically significant difference from each other, using Tukey’s test (*p* < 0.05); Table S3: Mechanical properties of BM before and after 12 h immersion in SBF: Young’s modulus (MPa) with statistically significant difference from each other, using Tukey’s test (*p* < 0.05); Table S4: Mechanical properties of BM before and after 12 h immersion in SBF: Stiffness (N/m) with statistically significant difference from each other, using Tukey’s test (*p* < 0.05); Table S5: Mechanical properties of BM before and after 12 h immersion in SBF: Tensile Strength (MPa) with statistically significant difference from each other, using Tukey’s test (*p* < 0.05).
