# Supplementary material for: Electrospun Polycaprolactone Membranes Loaded with Gentamicin and Nano-Hidroxyapatite for Guided Bone Regeneration
Source: Biomedicines. 2025 Sep 25;13(10):2349. doi: 10.3390/biomedicines13102349 (PMC12561880; doi:10.3390/biomedicines13102349)
Supplement: Supplementary file 1 [file biomedicines-13-02349-s001.zip › biomedicines-3689761-supplementary.pdf]

Supporting Information

for Biomedicines

Electrospun Polycaprolactone Membranes Loaded with Gentamicin and Nano-Hidroxyapatite for Guided Bone Regeneration

Ioana-Codruta Mirica [1], Gabriel Furtos [2,*], Véronique Fontaine [3], Mihaela Vlassa [2,*], Petru Pascuta [4], Ioan Petean [5], Bogdan Bâldea [6], Otilia Andercou [7] and Ondine Patricia Lucaciu [1]

[1] Department of Oral Health, Iuliu Hatieganu University of Medicine and Pharmacy, 400012 Cluj-Napoca, Romania; mirica_codruta@yahoo.com (I.-C.M.); ondineluc@yahoo.com (O.L.)
[2] Department of Dental Materials, Raluca Ripan, Institute of Research in Chemistry, Babes-Bolyai University, 400294 Cluj-Napoca, Romania
[3] Faculty of Pharmacy, Microbiology, Bioorganic and Macromolecular Chemistry Unit, Université libre de Bruxelles (ULB), 1050, Belgium; veronique.fontaine@ulb.be (V.F.)
[4] Department of Physics and Chemistry, Technical University of Cluj-Napoca, 400641 Cluj-Napoca, Romania; petru.pascuta@phys.utcluj.ro (P.P.)
[5] Faculty of Chemistry and Chemical Engineering, Babes-Bolyai University, 400028 Cluj-Napoca, Romania; petean.ioan@gmail.com (I.P.)
[6] Department of Prosthodontic Dentistry, Nicolae Testimiteanu, State University of Medicine and Pharmacy, 2004 Chisinau, Moldova; bogdanbaldea@gmail.com (B.B.)
[7] Radiobiology and Tumor Biology Department, Oncology Institute Prof. Dr. I. Chiricuţă, 400015 Cluj-Napoca, Romania; otiliabarbos2006@yahoo.com (O.A)
* Correspondence: gfurtos@yahoo.co.uk (G.F.); mihaela_cecilia@yahoo.com (M.V.)

Table S1. Fiber diameter from SEM images of BM, with statistically significant difference from each other, using Tukey's test (*p < 0.05*).

Nr.	Samples	Fiber diameter (μm) (SD)
1	PCL	2.55 (0.42) a
2	PCL-5%nHAP	3.28 (0.89) b
3	PCL-10%nHAP	3.9 (1.56) a, c
4	PCL-15%nHAP	4.21 (1.26) a, c
5	PCL-0.5%GEN	2.64 (0.66) c, d
6	PCL-1%GEN	2.89 (0.92) c, e, f, g
7	PCL-2%GEN	4 (1.12) a, d, g, h
8	PCL-5%nHAP-0.5%GEN	4.09 (1.21) a, d, e
9	PCL-5%nHAP-1%GEN	4.09 (2.24) a, d, g
10	PCL-5%nHAP-2%GEN	4.15 (1.32) a, d, g, h
11	PCL-10%nHAP-0.5%GEN	5.78 (2.6) a, b, c, d, e
12	PCL-10%nHAP-1%GEN	5.05 (1.91) a, b, c, d, g, h
13	PCL-10%nHAP-2%GEN	5.45 (1.29) a, b, c, d, e, f, g, h
14	PCL-15%nHAP-0.5%GEN	4.27 (1.73) a, f
15	PCL-15%nHAP-1%GEN	5.04 (1.57) a, b, c, d, g
16	PCL-15%nHAP-2%GEN	5.17 (1.44) a, b, c, d, e, g, h

Note: SD, standard deviation; letters (a-h) right to values indicate within column mean values with statistically significant differences using Tukey's test (* $p < 0.05$)

Table S2. Mechanical properties of BM before and after 12 h immersion in SBF: Force at maximum load (N) with statistically significant difference from each other, using Tukey's test ($p < 0.05$).

Nr.	Samples	Load at Maximum Load (N) (SD)	
		Before immersion in SBF	After immersion in SBF
1	PCL	1.18 (0.05)a	1.05 (0.21)b
2	PCL-5%nHAP	1.7 (0.22)c	1.53 (0.19)b,d
3	PCL-10%nHAP	1.2 (0.13)a,b,c,e	1.14 (0.21)a,b,c,f
4	PCL-15%nHAP	1.34 (0.22)a,b,g	1.08(0.17),b,e,f,h
5	PCL-0.5%GEN	3.52 (0.98)a,b,g,i	2.63 (0.51)d,e,f,g,h,i,j
6	PCL-1%GEN	3.24 (0.29)b,e,f,i,k	2.31 (0.11)b,f,l
7	PCL-2%GEN	2.99 (0.15)f,g,j,m	2.64 (0.59)e,f,g,i,n
8	PCL-5%nHAP-0.5%GEN	2.48 (0.75)d,e,f,h,i,l,m,o	2.06 (0.22)e,f,i,p
9	PCL-5%nHAP-1%GEN	2.61 (0.52)e,f,g,i,q	1.15 (0.37)e,f,g,i,r
10	PCL-5%nHAP-2%GEN	1.93 (0.14)e,f,i,s,	1.47 (0.22)a,b,c,h,j,k,l,m,n,o,p,q,r,s,t
11	PCL-10%nHAP-0.5%GEN	2.09 (0.22)d,e,f,g,h,i,l,m,t,u	1.06 (0.19),b,g,i,j,o,s,t,u,v
12	PCL-10%nHAP-1%GEN	1.68 (0.37)c,d,e,f,h,i,k,l,m,n,p,q,r,s,t,v,w	1.55 (0.36)d,e,f,h.i,l,m,t,v,x
13	PCL-10%nHAP-2%GEN	1.66 (0.34)d,e,f,g,h,i,l,m,r,s,t,v,y	1.33 (0.25)c,d,e,f,g,h,i,m,p,r,t,v,z
14	PCL-15%nHAP-0.5%GEN	3.24 (0.29)d,e,f,h,i,m,t,v,A	0.88 (0.25)e,f,h,i,t,v,w,B
15	PCL-15%nHAP-1%GEN	2.19 (0.48)d,e,f,g,h,i,l,m,r,t,v,C	0.49 (0.15)a,b,c,d,g,h,j,k,l,n,o,p,q,r,s,t,u,v,w,x,y,z,A,B,C
16	PCL-15%nHAP-2%GEN	0.83 (0.12)a,c,j,k,n,o,p,q,s,u,w,x,y,z,A,B,C	1.03 (0.14)a,b,c,h,j.k,l,m,n,o,p,q,s,u,w,x,y,z,A,B,C

Note: SD, standard deviation; letters (a-C) right to values indicate within column mean values with statistically significant differences using Tukey's test (* $p < 0.05$)

Table S3. Mechanical properties of BM before and after 12 h immersion in SBF: Young's modulus (MPa) with statistically significant difference from each other, using Tukey's test ($p < 0.05$).

Nr.	Samples	Young's Modulus (MPa) (SD)	
		Before immersion in SBF	After immersion in SBF
1	PCL	13.96 (4.25)a,	6.52 (4.25)
2	PCL-5%nHAP	7.36 (3.5)	4.65 (3.5)b
3	PCL-10%nHAP	11.05 (5.55)a,c	4.71 (5.55)a,d
4	PCL-15%nHAP	9.78 (2.78)a,e	5.04 (2.78)a,f
5	PCL-0.5%GEN	4.65 (1.62)a,g	4.12 (1.59)a,h
6	PCL-1%GEN	5.27 (2.89)a,i	4.57 (0.75)a,j
7	PCL-2%GEN	7.85 (1.75)a,k	3.85 (0.9)a,l
8	PCL-5%nHAP-0.5%GEN	4.23 (1.18)a,b,m	3.28 (0.71)a,n
9	PCL-5%nHAP-1%GEN	3.77 (1.64)a,c,o	3.61 (1.37)a,p
10	PCL-5%nHAP-2%GEN	4.97 (1.22)a,q	3.29 (1.08)a,r
11	PCL-10%nHAP-0.5%GEN	3.2 (1.13)a,s	3.02 (0.37)a,t
12	PCL-10%nHAP-1%GEN	6.44 (1.63)a,u	2.49 (1.02)a,v
13	PCL-10%nHAP-2%GEN	4.97 (1.04)b,d,e,f,g,h,i,j,k,l,m,n,o,p,q,r,s,t,u,v,w	4.24(0.77)a,w
14	PCL-15%nHAP-0.5%GEN	4.57 (0.75)a,w,x	3.62 (1.03)d,e,f,g,h,i,,j,k,l,m,n,o,s,t,u
15	PCL-15%nHAP-1%GEN	4.21 (3.89)a,w	3.14 (0.53)a,w
16	PCL-15%nHAP-2%GEN	5.33(3.53)a,w,x	4.74 (1.38)a,w

Note: SD, standard deviation; letters (a-x) right to values indicate within column mean values with statistically significant differences using Tukey's test (* $p < 0.05$)

Table S4. Mechanical properties of BM before and after 12 h immersion in SBF: Stiffness (N/m) with statistically significant difference from each other, using Tukey's test (*$p < 0.05$*).

Nr.	Samples	Stiffness (N/m) (SD)	
		Before immersion in SBF	After immersion in SBF
1	PCL	1668.71 (264.35)a	1044.73 (296.51)
2	PCL-5%nHAP	1079.3 (387.83)	921.03 (221.71)
3	PCL-10%nHAP	1366.59 (371.58)	932.23 (167.76)
4	PCL-15%nHAP	1256.1 (408.07)	995.25 (249.93)
5	PCL-0.5%GEN	1079.3 (387.83)	845.59 (355.07)a,b
6	PCL-1%GEN	1191.84 (306.55)	989.89 (541.37)a
7	PCL-2%GEN	1231.16 (235.97)	977.87 (211.71)
8	PCL-5%nHAP-0.5%GEN	1378.23 (549.66)	785.73 (154.94)
9	PCL-5%nHAP-1%GEN	1054.19 (509.31)	552.21 (227.58)
10	PCL-5%nHAP-2%GEN	890.13 (256.06)	593.45 (149.01)
11	PCL-10%nHAP-0.5%GEN	788.14 (271.14)	830.04 (47.35)
12	PCL-10%nHAP-1%GEN	1137 (313.39)	631.41 (257.67)
13	PCL-10%nHAP-2%GEN	991.4 (559.6)b	921.51 (209.73)
14	PCL-15%nHAP-0.5%GEN	1191.84 (306.55)	660.64 (170.72)
15	PCL-15%nHAP-1%GEN	860.19 (179.91)	620.37 (536.86)
16	PCL-15%nHAP-2%GEN	636.03 (236.59)	607.63 (419.37)

Note: SD, standard deviation; letters (a-b) right to values indicate within column mean values with statistically significant differences using Tukey's test (* $p < 0.05$)

Table S5. Mechanical properties of BM before and after 12 h immersion in SBF: Tensile Strength (MPa) with statistically significant difference from each other, using Tukey's test (*$p < 0.05$*).

Nr.	Samples	Tensile Strength (MPa) (SD)	
		Before immersion in SBF	After immersion in SBF
1	PCL	1.64 (0.2)a	1.08 (0.43)b
2	PCL-5%nHAP	1.67 (0.48)c	0.86 (0.08)a,c,d
3	PCL-10%nHAP	1.15 (0.19)b,d,e	0.71 (0.11)f
4	PCL-15%nHAP	1.26 (0.36)a,c,e,f,g	0.65 (0.07)a,c,e,h
5	PCL-0.5%GEN	1.51 (0.24)a,c,e,i	0.86 (0.08)a,c,e,j
6	PCL-1%GEN	1.5 (0.26)e,k,	1.08 (0.37)a,e,l
7	PCL-2%GEN	1.82 (0.3)a,c,e,m	1.38 (0.11)a,c,e,f,n
8	PCL-5%nHAP-0.5%GEN	1.02 (0.17)a,c,d,e,f,g,i,k,o	0.78 (0.11)a,c,e,o,p
9	PCL-5%nHAP-1%GEN	1.12 (0.25)a,c,e,f,q	0.89 (0.28)a,c,e,f,r
10	PCL-5%nHAP-2%GEN	1.25 (0.14)a,c,e,f,k,s	0.98 (0.16)a,c,h,j,l,m,n,o,q,r,s,t
11	PCL-10%nHAP-0.5%GEN	0.89 (0.06)a,c,d,e,f,i,t,u	0.51 (0.07)a,c,e,t,v
12	PCL-10%nHAP-1%GEN	1.02 (0.14)a,b,c,d,e,f,g,i,j,k,m,p,t,v,w	0.78 (0.14)a,c,e,f,l,t,x
13	PCL-10%nHAP-2%GEN	0.84 (0.14)e,o,u,w	0.69 (0.19)a,c,e,f,k,t,y
14	PCL-15%nHAP-0.5%GEN	1.5 (0.26)a,c,e,f,k,t,z	0.56 (0.12)e,o,s,u,w,x,y,z,A
15	PCL-15%nHAP-1%GEN	0.94 (0.16)a,c,e,f,k,t,A,B	0.37 (0.11)h,j,l,m,n,o,q,r,s,u,v,w,x,y,z,B
16	PCL-15%nHAP-2%GEN	0.77 (0.3)a,c,e,f,t	0.66 (0.08)h,j,l,m,n,q,r,s,u,v,w,x,y,z,B

Note: SD, standard deviation; letters (a-B) right to values indicate within column mean values with statistically significant differences using Tukey's test (* $p < 0.05$)
